# Emergency airway management in resource limited setting

**DOI:** 10.1186/s12245-024-00607-3

**Published:** 2024-03-14

**Authors:** Gbolahan Olatunji, Emmanuel Kokori, Nicholas Aderinto, Mohammed Alsabri Hussein Alsabri

**Affiliations:** 1 Department of Medicine and Surgery, University of Ilorin, Ilorin, Nigeria; 2 Department of Medicine and Surgery, Ladoke Akintola University of Technology, Ogbomoso, Nigeria; 3 Althawara Moderen General Hospital, Sanaa, Yemen

**Keywords:** Emergency airway management, Resource-limited settings

## Abstract

**Background:**

Emergency airway management in resource-limited settings presents multifaceted challenges due to shortages in essential medical resources, healthcare professionals, and infrastructure.

**Methods:**

We conducted a literature search using keywords “Emergency Airway Management” “Low Resource” “Africa” “Asia” from databases such as Pubmed, and Google Scholar, from where we extracted relevant literature for our study.

**Findings:**

These limitations resulted in delayed interventions, suboptimal care, and higher complication rates during intubation procedures. However, innovative solutions have emerged to address these challenges, including cost-effective airway management devices and training programs tailored for non-medical personnel. Capacity building and local empowerment are critical components of improving emergency airway management in these settings. Additionally, advocating for policy support and investment in healthcare infrastructure is essential to ensure access to essential equipment and adequate staffing. Collaboration and knowledge-sharing networks among healthcare professionals and organisations are pivotal in disseminating best practices and advancing healthcare delivery in resource-limited regions.

**Conclusion:**

Future efforts should focus on tailored training programs, rigorous research, innovative device development, telemedicine solutions, sustainable capacity building, and advocacy to enhance emergency airway management in resource-limited settings.

## Introduction

Airway management is fundamental to medical practice, particularly in emergency medicine. However, when faced with resource-limited settings defined as countries with a GNI per capita of $1,135 or less for low-income countries and a range of $1,136 to $4,465 for lower-middle-income countries [1]. Making up approximately 63% of world countries [2], these settings are often characterised by a shortage of conventional tools and expertise with implications for healthcare providers in providing effective airway management during emergencies [3]. Emergency airway management is a critical life-saving procedure for patients experiencing various acute conditions, where swiftly and effectively securing the airway takes precedence [4]. Approximately 0.5–1% of patients presenting in emergency departments (EDs) are estimated to require intubation due to critical conditions such as respiratory failure, cardiac arrest, and altered mental status [5, 6]. Among these patients, approximately 10% encounter the challenge of difficult intubation, a percentage that may rise notably in resource-limited settings [7–9]. Difficult intubation arises in situations where there is a challenging airway, defined as a scenario in which a proficient healthcare provider anticipates or encounters difficulty with any or all of the following: face mask ventilation, direct or indirect (e.g., video) laryngoscopy, tracheal intubation, supraglottic device (SGD) utilisation, or surgical airway procedures [10].

Intriguingly, endotracheal intubation in the emergency room carries a 12% complication risk, with adverse outcomes ranging from oesophagal intubation and mainstem intubation to hypotension and cardiac arrest [3].

Emergency airway management within resource-limited settings introduces various challenges that necessitate innovative solutions. Unlike well-equipped medical facilities, these settings often grapple with shortages of essential medical resources, including intubation equipment, medications, and skilled personnel [11–13]. In resource-constrained environments, conventional approaches to airway management may prove impractical or suboptimal. This constraint compels healthcare providers in such settings to devise creative strategies or enhance outdated methods to achieve comparable results.

The significance of training emergency specialists cannot be overstated in meeting the demands of airway management within resource-limited settings. Notably, in Sub-Saharan Africa as an example, a region characterised by resource constraints, specialised emergency medicine training programs are notably scarce. As of 2016, only seven universities in the entire region had successfully graduated emergency medicine (EM) specialists, with four of these institutions located in South Africa [14]. This region is home to a population of 1.1 billion people, constituting roughly 14% of the world’s population [15]. Pakistan is another illustrative example; the country initiated its first emergency medicine training program in 2010, and as of January 2020, nine institutions within Pakistan offer EM speciality training programs. However, the specialist density remains relatively low at approximately 0.02 physicians per 100,000 people [16].

The scarcity of specialist training emphasises the critical necessity for healthcare personnel to cultivate innovative and adaptive methods for emergency airway treatment in these challenging areas. When faced with a scarcity of readily available knowledge, medical personnel in resource-constrained environments usually rely on their creativity and innovative problem-solving skills to address various airway management difficulties. This frequently leads to creating unique, creative solutions carefully tailored to solve the particular challenges experienced in these contexts [17, 18].

These efforts have resulted in remarkable advancements, such as developing and deploying oxygen concentrators tailored for use in low-resource situations. These concentrators are a reliable and cost-effective substitute for more expensive oxygen plants and piped systems [18].

In light of these considerations, this review explores the current literature concerning emergency airway management in resource-limited settings. Its primary objectives are to highlight the existing evidence, provide insights into the current landscape of training for emergency physicians in these settings, specify the equipment utilised, delve into the various approaches to airway management for both adult and pediatric patients, and outline the contemporary challenges and prospective avenues for future advancement.

## Methodology

A comprehensive search of peer-reviewed literature was conducted utilising reputable databases, including PubMed, Scopus, Web of Science, and Google Scholar. A combination of relevant keywords and Medical Subject Headings (MeSH) terms was utilised to identify studies. Keywords included “emergency airway management,” “resource-limited settings,” “airway management techniques,” “alternative airway devices,” and related terms.

The criteria for article inclusion centred on studies published in English within the last two decades, specifically targeting emergency airway management within resource-limited settings. Included studies encompassed a range of research types, including original investigations, reviews, and case studies conducted within resource-limited environments.

Two independent reviewers screened the studies based on the inclusion criterion. The selection process prioritised articles the authors deemed to make unique and substantial contributions to the topic. Exclusion criteria were judiciously applied to filter out studies unrelated to the core subject matter, especially those dealing with airway management in well-resourced settings or non-resource-limited populations.

**Fig. 1:**
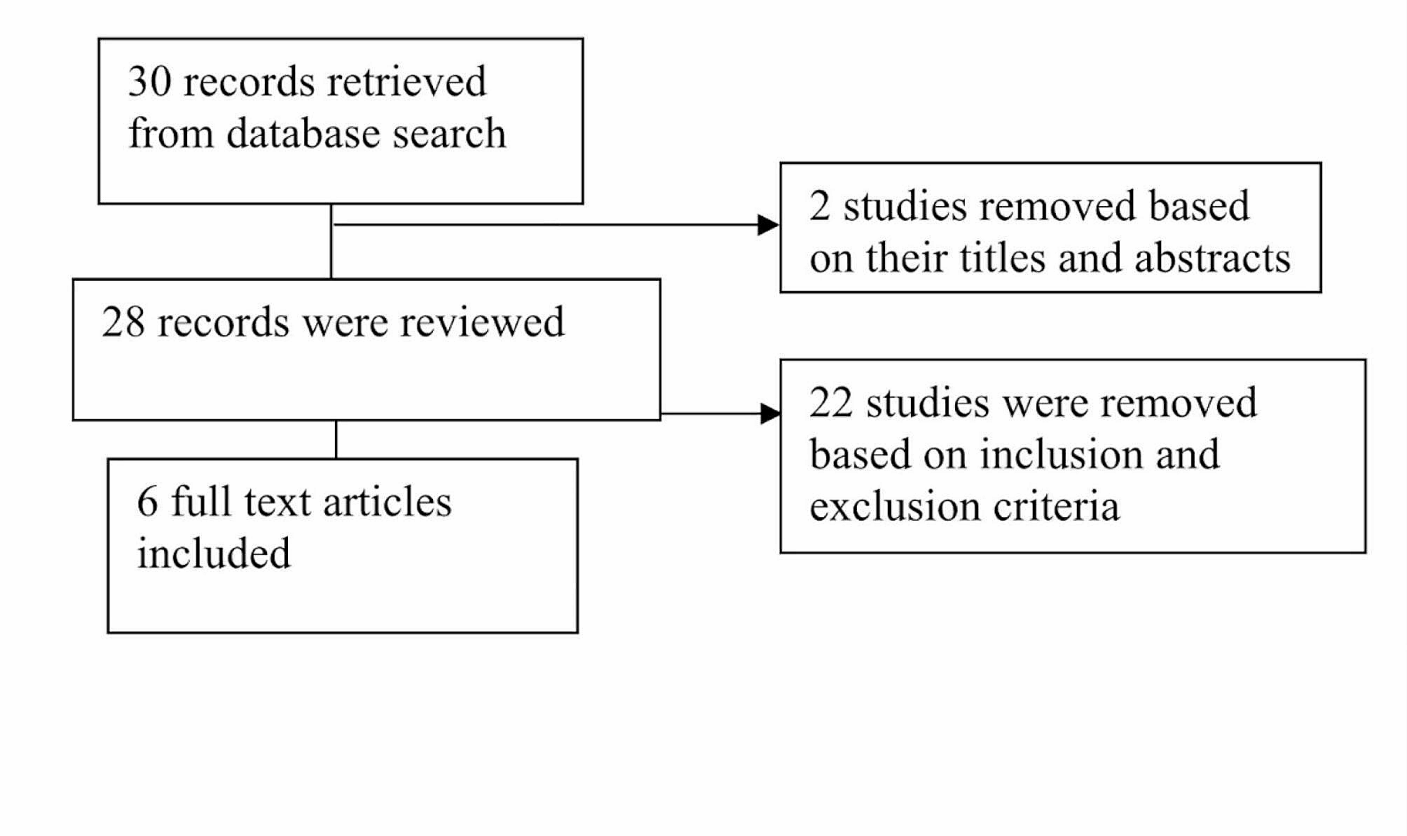
Flow chart showing the selection of studies

Two independent reviewers extracted data from the selected articles, emphasising key aspects such as airway management techniques, study design, patient demographics, intervention protocols, primary outcomes, and safety profiles. The review also discussed the challenges and innovations in emergency airway management within resource-limited healthcare settings. Any discrepancies were resolved through consensus or consultation with a third reviewer. Figure 1.

## Results

### Identification of studies

A total of 30 studies were identified through a comprehensive search of electronic databases. After a thorough screening process, six studies met the defined inclusion criteria and were used in this review ( Figure one ).

### Epidemiology and challenges

Training of skilled practitioners is paramount in enhancing emergency medical services like airway intubation, especially in low-resource settings with often severe shortages of such skilled personnel. For example, in a Western Cape facility in South Africa, emergency clinicians, including medical officers, registrars in emergency medicine, and general surgery, are trained in trauma cases [18]. With registrars rotating through, they are in charge of running the trauma resuscitation unit. All employees have received Advanced Trauma Life Support (ATLS) training, giving them the knowledge and abilities to manage trauma cases successfully. The attending physician protects patients’ airways, choosing the type and technique with care. There is a limited selection of equipment for restricted airways, although standard equipment and drugs are readily available. A team approach is used during intubation procedures, and precautions such as cricoid pressure are applied to stop reflux and aspiration. Tracheal intubation is clinically established through examination, auscultation, and observation. Approximately 61.4% of trauma patients survive, with a sizable minority necessitating surgery or critical care [19].

There have been efforts to improve skill and understanding of this crucial procedure, such as the Emergency Airway + Ventilation Course in Kenya [20]. It is a training course designed to give medical staff at African healthcare facilities the knowledge, abilities, and attitudes required to intubate and ventilate critically ill patients safely. The course discusses deficiencies in emergency airway treatment and ventilation, including insufficient supplies and qualified medical personnel. It is designed to satisfy the demands of nurses, clinical officers, and medical officers working in emergency rooms and intensive care units. Pre-course instruction, assessments, workshops, evaluations of clinical abilities, and a post-test are all included in the course. The ability to complete the course will show competence in patient management, airway management, and oxygen delivery equipment. Similar is the Kochi advanced airway management workshop in India [21].

Successful first intubation in resource-limited settings relies on various critical factors. Clinical expertise is paramount, with experienced healthcare providers essential for swift and effective intubation. Patient assessment is crucial in identifying potential airway challenges like facial trauma or anatomical abnormalities. Access to functional intubation equipment, including laryngoscopes and endotracheal tubes, is vital [19].

### The approach to difficult airway in low resource settings and success rates

Hardcastle’s observational study demonstrates a unique airway management method [19]. In this resource-constrained environment, emergency medicine registrars and medical officers largely carried out definitive airway management operations. Notably, emergency physicians with a variety of training used the recommended technique, rapid sequence intubations (RSIs). Five to six per cent of the 57 trauma patients who needed definitive airway care were intubated on-site, and all of them used RSIs. Paramedics did 12.3% of pre-hospital intubations, whereas 31.6% were done by non-anesthetists at the referral institutions. The success rate of endotracheal intubation was 96.4%, and only 3.6% of patients required a surgical cricothyroidotomy. The most common reason for intubation (52.6%) was a Glasgow Coma Score (GCS) of less than 8, which is frequently seen in patients with polytrauma from car accidents. This is comparable to a 91% first pass and 99% intubation success rate in a Finnish pre-hospital emergency setting [22]. It is substantially higher than the 88.6% and 76.5% recorded in a tertiary hospital in India using the King Vision video laryngoscope (KVVL) and Macintosh laryngoscope respectively which are deemed more sophisticated and costlier systems [23].

Similarly, Saoraya et al. ‘s retrospective study emphasises how common difficult airway predictors are, calling for careful assessment before emergency airway treatment. Patients with at least one problematic airway predictor received sedation and neuromuscular blocking medications in 57.4% of cases, compared to 35.5% who simply received sedation [24]. First-pass success was attained in 74.3% of patients, with no discernible differences between those with and without predictors in terms of glottic view, first-pass success, or complications. Notably, the study points out the drawbacks of utilising the Look–Evaluate–Mallampati– Obstruction–Neck mobility components (LEMON) criteria to forecast unsuccessful glottic views and first passes.

In addition, the four-year prospective observational study by Evelyn Wong et al., which includes 1068 cases, gives a thorough picture of patients needing emergency airway treatment [21]. Emergency physicians with a range of training primarily used orotracheal intubation with variable drug regimes. Surprisingly, 99.6% of orotracheal intubations were successful overall. The study emphasises emergency physicians’ important and safe role in managing airways in low-resource settings.

Shauer et al. explored battlefield scenarios where airway compromise is a potential concern. Despite challenging conditions, there were no substantial differences in short-term outcomes between casualties who received Supraglottic airway (SGA) placement and those who underwent cricothyrotomy [24]. This suggests that even in high-stress, resource-constrained military prehospital environments, low-resource alternatives like SGA placement can be considered viable options for airway management.

Equally, the study by Ayalew et al. at an Ethiopian facility provides information on the success rates and risks connected with emergency intubations in this area. Out of 15,933 patients seen in the department for a year, 256 (1.6%) required intubation [25]. The primary reasons for intubation were respiratory failure management and airway protection. Intubation procedures varied regarding anaesthesia administration, with sedation-only intubation being preferred for patient comfort and rapid sequence intubation being used in many cases. A smaller subset of patients underwent intubation without anaesthesia, indicating further exploration of clinical implications. The success rates of intubation attempts were high, with most patients (70.3%) achieving successful first-pass intubation. Second-pass intubation was successful in cases where first-pass attempts failed. The medical team’s competence in navigating airway management complexities was evident, even in situations requiring up to three attempts. However, a major challenge during these procedures was hypoxia, characterised by inadequate oxygen levels. Further investigation into factors contributing to hypoxia could help refine airway management strategies and optimise patient outcomes.

In Malaysia, Shahridan Mohd et. Al’s study provides information about airway management in a university emergency room. Two hundred twenty-eight intubations were included in this study [26]. Cardiopulmonary arrest (35.5%), head injury (18.4%), respiratory failure (15.4%), polytrauma (9.6%), and cerebrovascular accident (7.0%) were the main causes of intubation. All 228 patients were successfully intubated; quick sequence intubation was used the most frequently (49.6%). It’s noteworthy that 79.8% of intubations were successful the first time. The study offers insights into the usage of induction agents, with suxamethonium serving as the preferred muscle relaxant and midazolam serving as the most popular induction agent. Even though they were documented in 14.9% of instances, oesophageal intubation largely caused immediate problems.

M Wongyingsinn et al. at Siriraj Hospital revealed that 7 people received endotracheal intubation over a year [27]. Notably, 79.5% of these intubations were effective on the first try, making 99.6% successful [11]. Neuromuscular blocking drugs were only necessary in a limited number of patients, and they were only given by anesthesiologists. 68.1 per cent of patients underwent intubation without the use of any medications.

Another study that took place in a tertiary care facility in India for 16 months throws insight into important elements of emergency airway management. Notably, it demonstrates the rapid sequence intubation (RSI) technique’s ubiquity in this situation by showing that emergency physicians largely used it in 72.9% of instances [28]. The study had a first-pass success rate of 78.3%. The fact that emergency medicine (EM) residents perform orotracheal intubation for all patients without the need for surgical airways also emphasises the significance of specialised training in emergency airway management and the critical role that properly trained personnel play in ensuring efficient and effective emergency intubation procedures.

Difficult airway intubation is not exempted of adverse events, especially in low resource settings, Anudeep et al. reported an incidence of airway injury in 32.8% of patients intubated by non-anaesthetic residents and a much lower 5.9% amongst patients intubated by residents undergoing anaesthesiology training [29]. This exemplifies how inadequate training in several low-resource centres can complicate the procedure. For example, in Nigeria, there are about 1200 anaesthesiologists to cater for a population of over 200 million [30]. Similarly, Ayalew et al. reported complication rates of almost 30%, with hypoxia, hypotension and bradycardia accounting for roughly two-thirds of the complications [25].

As shown above, Intubation in low-resource settings remains a complex issue that can substantially impact patient outcomes and emergency airway management effectiveness. The primary issue is the limited availability of essential equipment, such as laryngoscopes, endotracheal tubes, and medications, which can lead to improvisation and reliance on alternative techniques. Variability in the qualifications of healthcare providers, including non-anesthetists, can also lead to inconsistencies in technique and decision-making. The lack of continuous education and standardised training programs can also hinder healthcare providers’ ability to keep up with evolving best practices. Access to courses like Advanced Trauma Life Support (ATLS) and Emergency Airway + Ventilation Course can be limited, affecting the quality of care provided.

Medication availability, particularly for induction and paralysis during intubation, can also be limited due to variability in drug choices. The higher volume of trauma cases in these settings necessitates preparedness for difficult airways, but the limited availability of advanced tools can hinder securement. Adverse events like desaturation, bronchial intubation, and equipment failures are common, underscoring the need for rigorous monitoring and quick interventions. Addressing these challenges requires improving equipment access, standardising training, and enhancing overall preparedness to ensure patient outcomes in resource-constrained environments.

## Discussion and future directions

Emergency airway management in low-resource countries is marked with challenges, each profoundly impacting healthcare delivery within these regions. These regions frequently lack access to advanced medical equipment for effective airway management [3]. Vital apparatuses, such as endotracheal tubes, laryngoscopes, ventilators, and monitoring devices, are often in short supply or absent—the shortage of such equipment results in delayed and suboptimal interventions during critical airway-related medical emergencies. In addition, the acute shortage of adequately trained healthcare professionals specialising in airway management perpetuates this challenge [31]. Anesthesiologists, respiratory therapists, and nurses skilled in airway procedures are frequently scarce commodities. The scarcity of such professionals hampers the timely and proficient execution of airway interventions, further exacerbating the difficulties faced in these settings. Infrastructural limitations are also an endemic concern [32]. Low-resource countries deal with insufficient healthcare infrastructure, such as inadequate hospital facilities, erratic electricity supply, and unreliable medical supply chains [33]. These infrastructural constraints impede the efficient maintenance and utilisation of airway management equipment, impinging upon the overall quality of care.

However, amid these challenges, innovation and adaptive strategies have emerged, tailored specifically to the difficulties of low-resource environments. Innovations in the form of cost-effective airway management devices have materialised, designed to operate efficiently in resource-constrained contexts. Examples include supraglottic airway devices and manual ventilation tools, offering alternatives to prohibitively expensive equipment and addressing the equipment shortage challenge [34, 35]. Moreover, recognising the scarcity of trained healthcare professionals, select organisations have initiated training programs tailored for non-medical personnel [5]. These programs, catering to community health workers and first responders, provide essential training in airway management techniques, thereby empowering non-medical personnel to administer initial care until professional assistance becomes available. Capacity building is integral to improving emergency airway management in low-resource settings. Successful training programs have empowered local healthcare workers, equipping them with the competencies and knowledge to manage airway emergencies adeptly.

## Conclusions and recommendations

It is imperative to allocate substantial resources towards developing and implementing tailored training programs designed explicitly for healthcare workers in low-resource countries. These programs should be meticulously structured to address the unique challenges of airway management in these settings. Emphasis should be placed on imparting fundamental airway management competencies, and equipping healthcare personnel with the requisite skills to respond effectively to emergencies.

A commitment to rigorous research is paramount to establishing evidence-based airway management practices attuning to the nuanced demands of low-resource settings. These research initiatives should encompass comprehensive assessments of the efficacy and suitability of various airway devices and protocols within resource constraints. A robust data collection framework should be established to capture critical insights and outcomes.

Fostering a conducive environment for developing and disseminating cost-effective, durable, and easily maintainable airway management devices tailored to low-resource environments is paramount. By encouraging innovation in this sphere, we can address the equipment scarcity challenge and enhance the accessibility of critical tools for airway interventions [36].

Exploring and adopting telemedicine and digital health solutions for remote guidance in airway management should be actively pursued. These technologies have the potential to bridge geographical gaps, enabling expert assistance and guidance to be delivered promptly to healthcare providers in remote or underserved areas.

Sustainable capacity building should be promoted through the empowerment of local healthcare workers. These individuals should be groomed to assume leadership roles in education and training, thereby ensuring the perpetuation of skill development initiatives. Establishing enduring partnerships with local institutions and organisations is instrumental in this endeavour.

Robust advocacy efforts are required to garner policy support and financial investment towards the augmentation of healthcare infrastructure in low-resource countries. Particular emphasis should be placed on fortifying emergency airway management capabilities, including procuring essential equipment and providing adequate staffing.

Cultivating knowledge-sharing networks among healthcare professionals, researchers, and relevant organisations actively engaged in emergency airway management within low-resource settings is essential. These collaborative initiatives serve as vital conduits for disseminating best practices, exchanging insights, and furthering the overarching mission of enhancing healthcare delivery in these regions.

## Data Availability

Clinical data are available from the corresponding author but only on reasonable request.
